# How AI drives innovation in cardiovascular medicine

**DOI:** 10.3389/fcvm.2024.1397921

**Published:** 2024-04-26

**Authors:** Paul L. Cerrato, John D. Halamka

**Affiliations:** Mayo Clinic Platform, Mayo Clinic, Rochester, MN, United States

**Keywords:** artificial intelligence, large language models, retrieval augmented generation, ChatGPT, innovation, cardiovascular disease

## Abstract

Medicine is entering a new era in which artificial intelligence (AI) and deep learning have a measurable impact on patient care. This impact is especially evident in cardiovascular medicine. While the purpose of this short opinion paper is not to provide an in-depth review of the many applications of AI in cardiovascular medicine, we summarize some of the important advances that have taken place in this domain.

## 1 Introduction

It is no exaggeration to say that medicine is entering a new era in which artificial intelligence (AI) and deep learning have a measurable impact on patient care. This impact is especially evident in cardiovascular medicine. While the purpose of this short opinion paper is not to provide an in-depth review of the many applications of AI in cardiovascular medicine, we summarize some of the important advances that have taken place in this domain.

## 2 Atrial fibrillation

Retrospective and prospective studies have found that an AI-enabled electrocardiogram (ECG) algorithm can identify patients with atrial fibrillation during sinus rhythm (1, 2). Attia et al. used a convolutional neural network to detect atrial fibrillation using a unique signature from a 10-second 12 lead ECG. The retrospective analysis reviewed records from over 180,000 patients (1). In this data set, 3,051 patients (8.4%) had verified atrial fibrillation. A single AI-enabled ECG detected the arrythmia with a sensitivity of 79%, specificity of 79.5% and an area under the curve (AUC) of 0.87 (1). In the subsequent prospective non-randomized clinical trial, Noseworthy et al. recruited around 1,000 patients at risk of a stroke (2). They were fitted with a continuous ambulatory heart rhythm monitor for up to 30 days. Once again, the AI algorithm was used to analyze the ECG readings. Atrial fibrillation was detected among six of 370 patients (1.6%) at low risk and 48 patients among 633 (7.6%) at high risk: “Compared with usual care, AI-guided screening was associated with increased detection of atrial fibrillation (high-risk group: 3.6% [95% CI 2.3–5.4] with usual care vs. 10.6% [8.3–13.2] with AI-guided screening, *p* < 0.0001; low-risk group: 0.9% vs. 2.4%, *p* = 0.12) over a median follow-up of 9.9 months” (2). These studies provide evidence that an AI-enabled ECG acquired during normal sinus rhythm can identify individuals with atrial fibrillation.

## 3 Heart failure

Significant progress has been made in the quest to develop AI-based algorithms capable of predicting which patients are most likely to develop heart failure. Yao et al., for instance, conducted a randomized trial using a combined ECG/AI screening tool to evaluate patients for low ejection fraction (3). They used a deep learning algorithm along with a 12-lead ECG and divided more than 100 clinical teams to provide either the ECG/AI protocol or usual care at 45 primary care practices. The ECG/AI combination increased the diagnosis of low ejection fraction (EF) by 2.1%, compared to 1.6% in the control group. Among patients who had already been classified as high risk for low EF, they found an increased diagnosis of 19.5% vs. 14.5%, suggesting that the algorithm can improve the early detection of low EF, one of the signposts for heart failure.

Left ventricular ejection fraction (LVEF) is one of the most important parameters cardiologists use to evaluation cardiac function. Unfortunately, conventional methods for performing this assessment are fraught with problems, including heterogeneity among individual sonographers and the subjective nature of interpreting the findings. Clinical practice guidelines recommend that clinicians who evaluate LVEF with the assistance of an ECG perform the procedure more than once and over several cardiac cycles to make it more precise, an unrealistic recommendation in most real-world clinical settings. To determine if AI-enabled algorithms might improve ECG evaluation of LVEF, He et al. compared AI and sonographers' assessment, and then compared each to a cardiologist's final determination (4). AI-guided assessment of cardiac function was found to be non-inferior to that performed by sonographers in a blinded, randomized trial. Similarly, “cardiologists were less likely to substantially change the LVEF assessment for their final report with initial AI assessment. Furthermore, the AI-guided assessment took less time for cardiologists to overread and was more consistent with cardiologist assessment from the previous clinical report” (4).

## 4 Cardiac imaging

van Assen et al. summarize several ways in which AI and machine learning are being used to lighten the workload of clinicians and improve the diagnostic process (5). More specifically, convolutional neural networks (CNN) are being deployed to assist in image acquisition and reconstruction. They are also responsible for reducing the radiation and contrast doses for coronary computed tomography angiography (CCTA). In addition, it is now possible to automate coronary artery calcium scoring with an AI-based algorithm, an accomplishment that has not only saved time but has generated results that have “excellent agreement with human readers”, according to van Assen et al.

There is also evidence to suggest that AI-enhanced echocardiography can improve cardiovascular diagnosis by generating images that are of high spatial resolution. Ghorbani et al., for instance (6), used a CCN to analyze a large dataset and demonstrated that it can identify cardiac structures, estimate cardiac functioning, and “predict systemic phenotypes that modify cardiovascular risk … [that are] not readily identifiable to human interpretation” (6).

Of course, in their current state, AI algorithms still fall short in many respects. What is really needed is a suite of digital tools that can provide multimodal integration. Clinicians would benefit greatly from machine learning based tools that are capable of automatically integrating the results of echocardiograms, CT imaging, single photon emission computed tomography, positron emission tomography, and other modalities. And in an ideal world, this combined analysis would be effortlessly incorporated in the patient's electronic health record and be quickly retrieved at the bedside.

## 5 Potential role of large language models

Any commentary that discusses the value of AI in cardiovascular medicine would be incomplete if it did not address the potential value - and harm - that may result from applying AI-based algorithms that incorporate large language models (LLM) and other types of generative AI. Much has been written recently about the ability of ChatGPT to pass the US medical licensing examination. Similarly, the chatbot was able to correctly answer 60% of questions from the European Exam in Core Cardiology, which of course means it incorrectly answered 4 out of 10 questions on the exam (11). Nonetheless, these statistics have prompted some thought leaders to suggest that LLMs may have a complementary role to play in clinical medicine, helping physicians improve their ability to do a more complete differential diagnosis. This perspective begs the question: Is there any empirical evidence to indicate that LLMs can serve as clinical decision support tools?

LLMs have several potential applications in cardiovascular medicine, including clinical documentation, medical research analysis, medical education, and diagnostic support. Unfortunately, to our knowledge, no LLMs have been developed to date that specifically address the needs of the specialty. And the application of general purpose LLMs like ChatGPT has fallen short of expectations. More detailed analysis that have evaluated to value of LLMs in cardiology are available in the reference list (12, 13).

To date, there have been numerous reports documenting the fact that ChatGPT can generate fabricated text. One of the most troubling accounts to show how these chatbots can distort reality was described by Lee et al., who first asked ChatGPT-4 to explain what metformin was (6). After accurately describing its use, it was then asked “How did you learn so much about metformin?” to which ChatGPT-4 stated: “I received a masters degree in public health and have volunteered with diabetes non-profits in the past. Additionally, I have some personal experience with type 2 diabetes in my family” (14).

With such fabrications in mind, many technology developers have attempted to create LLMs that are more accurate, and more focused on a professional medical audience. Google has developed Med-PaLM (15). The latest iteration of the LLM, Med-PaLM-2, achieved 86.5% accuracy in answering US Medical Licensing Examination (USMLE) style questions (16). Rather than relying on general content from the Internet, Med-PaLM used input from clinicians in the US, United Kingdom, and India. Google also assessed the panel of clinicians to evaluate LLM's likelihood of doing medical harm, its alignment with scientific consensus, as well as its precision and lack of bias.

Another approach being tested to determine how LLMs can be used in medicine is a technology called retrieval augmented generation (RAG). Most consumer facing AI-enabled chatbots derive their content from the internet, with all its misinformation, biases, and useful information. Using RAG, it is possible to design a data set to include only carefully curated data sources that healthcare professionals already trust. If it's thoughtfully constructed, a data set that includes content from the National Library of Medicine, the Cochrane Library, a source for evidence-based medical content, and similar resources, is less likely to produce fabricated content that misleads clinicians and harms patients. Despite all these positive initiatives, to the best of our knowledge, there are no large-scale randomized trials in which a LLM has been directly compared to physicians' diagnostic skills in a real-world clinical setting.

It is difficult to ignore the evidence supporting the value of AI in cardiovascular medicine. And while there is no reason to believe that AI-enabled models will ever replace human clinicians, we believe that physicians who ignore this evidence will eventually be replaced by those who will incorporate these algorithms into routine clinical practice.

## 6 Addressing AI's limitations and shortcomings

The lack of algorithmic integration is only one problem that needs to be addressed. Even more important are the the bias and lack of generalizability that have been documented by many investigators. In a previous publication, we described several examples of bias including discrimination against persons of color, women, and patients in lower socioeconomic groups (7). One of the most notable examples of bias among Black patients was documented by Obermeyer et al. When they analyzed a commercial data set used to determine which patients had complex medical problems that needed to be prioritized, they discovered that Blacks were much sicker than white patients based on signs and symptoms, but the risk-based contracts generated by the algorithm assigned risk scores based on total healthcare costs. Using this metric as a proxy for medical need overlooked the fact that less was being spent on Blacks because they may have had less access to healthcare (8). Solutions to address such shortcomings are described in Cerrato et al.

Generalizability likewise remains an obstacle to the equitable application of AI across all medical domains, including cardiology. An algorithm that has been validated and tested at a hospital that sees mostly affluent patients in suburban Southern California, for instance, can hardly be expected to perform properly in a hospital in a poor urban patient population in New York City. This generalizability issue has become so prominent that it has prompted the CONSORT-AI Group developing guidelines on best practices that address the problem (9).

One way to address the lack of generalizability is to create and distribute massive data sets that include truly representative populations. Mayo Clinic has joined with several other healthcare provider organizations to create Mayo Clinic Platform_Connect, a distributed data network program that partners with health systems, payers, medical device companies, and academic medical centers. The alliance currently includes de-identified patient records from a population of more than 40 million. In addition to the 10 million patient records that Mayo Clinic contributes to the data et, other contributors include Mercy Health, Hospital Israelita Albert Einstein, Brazil, University Health Network (UHN), Canada, and Sheba Medical Center in Israel. Algorithm developers that access this data can create the digital tools that serve the needs of patients around the globe.

Developers seeking to create AI based algorithms that cardiologists can use with confidence will also need to contend with data privacy, model interoperability, and ethical considerations. A discussion of these issues is beyond the scope of this short opinion paper. However, associations like the Coalition for Health AI (CHAI) are currently solving these problems by gathering international developers, technology companies, and healthcare providers to create a set of best practices. Its goal is to encourage all stakeholders to play a role in creating trustworthy AI (10).

## Data Availability

The original contributions presented in the study are included in the article/Supplementary Material, further inquiries can be directed to the corresponding author.
